# Role of the Long Intergenic Non-Protein-Coding RNA 1278/miR-185-5p/Cystatin SN Axis in Laryngeal Cancer Cells

**DOI:** 10.1155/2022/6406943

**Published:** 2022-04-21

**Authors:** Bin Shen, Haibin Ding, Yanyan Ye, Baozhen Luo

**Affiliations:** Department of Otolaryngology, Shulan (Hangzhou) Hospital Affiliated to Zhejiang Shuren University, Shulan International Medical College, Hangzhou, Zhejiang, China 310015

## Abstract

Laryngeal cancer accounts for 25%–30% of tumors in the head and neck. Cystatin SN (CST1) was revealed to show upregulated expression in this cancer, while its functions and upstream pathway remain unknown and need investigation. The current study was designed to solve this problem. We designed short hairpin RNAs targeting CST1 for the loss-of-function assays to probe the influences of CST1 in laryngeal cancer cell proliferation and motility. The upstream competitive endogenous RNA pattern of CST1 was searched using bioinformatics analysis and confirmed by luciferase reporter assays. The experimental results demonstrated that CST1 is a tumor facilitator in laryngeal cancer by stimulating cellular proliferative, migrative, and invasive abilities. CST1 is regulated by the long intergenic non-protein-coding RNA 1278 (LINC01278)/miR-185-5p axis. LINC01278 knockdown and miR-185-5p overexpression exert the same functions as CST1 knockdown to repress cancer cell proliferation, migration, and invasion. In conclusion, LINC01278 plays an oncogenic role in laryngeal cancer by suppressing miR-185-5p to enhance CST1 expression, which enriches the molecular mechanism for the carcinogenesis of laryngeal cancer.

## 1. Introduction

Laryngeal cancer takes up 25%–30% of tumors in the head and neck [1]. Because of industrialization, aging, and high smoking rates, its incidence has increased [2]. Although there were advances in radiation, surgery, and chemotherapy for treatment of laryngeal cancer, the outcomes of patients with advanced cancer are poor [3]. The molecular mechanisms underlying laryngeal cancer tumorigenesis are not fully revealed. Lacking effective biomarkers hinders the diagnosis and therapy of laryngeal cancer. Thus, to explore the molecular mechanism for the tumorigenesis of laryngeal cancer possesses great importance.

Based on GSE84957, it was found that cystatin SN (CST1) is upregulated in 30 laryngeal cancer specimens compared with the adjacent nonneoplastic tissues by 4.256-fold changes [4]. CST1 is a member of the cystatin superfamily that suppresses the proteolytic activities of cysteine proteases [5]. In recent years, CST1 was found to be involved in the progression of various cancers, for instance, CST1 increases cell migrative and invasive capacities in gastric cancer [6]. It enhances cancer stem cell properties in papillary thyroid carcinoma and increases cell motility [7]. In colorectal cancer, CST1 mediates autophagy and oxidative stress response to control cell death [8]. These studies support the oncogenic role of CST1, while its functions in laryngeal cancer remain unclear. We hypothesized that CST1 can promote malignant phenotypes of laryngeal cancer cells and designed loss-of-function assays to explore it.

The competitive endogenous RNA (ceRNA) pattern is a classically posttranscriptionally regulatory mechanism in laryngeal cancer [9, 10]. Long noncoding RNAs (lncRNAs) with the length of more than 200 nucleotides and microRNAs (miRNAs) with the length of approximate 18-22 nucleotides are the dominant members in the ceRNA pattern [11]. A ceRNA network hypothesis has been proposed, which suggested that miRNAs play an important role in the ceRNA network by binding mRNA and inhibiting mRNA expression [12]. According to the ceRNA theory, lncRNAs can act as miRNA sponges regulating expression of the target mRNA by ceRNA network [13]. The discovery of these interactions presented a new perspective on cancer therapy. Although there are numerous studies on the functions of CST1 in various cancers [14, 15], its upstream ceRNA pathway has not been studied. We explored the upstream lncRNA and miRNA for CST1 in laryngeal cancer cells and investigated the functions of these noncoding RNAs, which might offer new insight into the mechanism of laryngeal cancer tumorigenesis.

## 2. Methods

### 2.1. Cell Culture

Laryngeal cancer cells TU686 (#MZ-2155, MINGZHOUBIO, Ningbo, China), M4E (#MZ-2348, MINGZHOUBIO), TU177 (#YS1198C, YaJi Biological, Shanghai, China), and TU138 (#MZ-4041, MINGZHOUBIO) cells and normal larynx epithelial-like cell line HuLa-PC (#CRL-3342, ATCC) were utilized in the present study. After proper resuscitation, cells were cultured in RPMI-1640 medium (GIBCO, USA) added with 12% fetal bovine serum in a humidified incubator at 37°C with 5% CO_2_. Every other day, the culture medium was changed.

### 2.2. Transfection

Short hairpin RNA (shRNA) targeting CST1 (sh-CST1) and LINC01278 (sh-LINC01278) and miR-185-5p mimics, designed by GenePharma (Shanghai, China), were utilized to reduce CST1/LINC01278 and increase miR-185-5p expression. To enhance CST1 functions, the CST1 cDNA was subcloned into the pcDNA3.1 vector (Invitrogen). TU686 and TU177 cells were transfected with the abovementioned plasmids or oligonucleotides at 22°C with the concentration of 50 nM using Lipofectamine 2000 (Life Technologies, Shanghai, China). After transfection for 5 h, the transfection medium was replaced. After 24 h, cells were harvested.

### 2.3. Reverse Transcription Quantitative Polymerase Chain Reaction (RT-qPCR)

Isolation of total RNA from whole-cell lysates was done using TRIzol reagent (Invitrogen). Cytoplasmic and nuclear parts of TU686 and TU177 cells were obtained using NE-PER Nuclear and Cytoplasmic Extraction Reagents (Thermo Fisher Scientific). Total RNA was reverse transcribed with a PrimeScript RT Reagent Kit (TaKaRa). PCR amplification was conducted using SYBR Premix Ex Taq II (TaKaRa). miR-185-5p was reverse transcribed with a Mir-X™ miRNA First Strand Synthesis Kit (TaKaRa), followed by amplification with a Mir-X™ miRNA RT-qPCR SYBR® Kit (TaKaRa). The thermal cycling condition for PCR is 95°C for denaturation, 55°C for annealing, and 65°C for elongation. The primer sequences (5′→3′) were listed as follows: CAST1, F, ATATGTACCAAGTCCCAGCC, R, AGAGCACAACTGTTTCTTCTG; miR-185-5p, F, TGGAGAGAAAGGCAGTTCCTG, R, CTCTACAGCTATATTGCCAGCCAC; LINC01278, F, CTGTCAGTCTTTGATGATGTCAG, R, TACCTTGCTTGGTCACTCAG; GAPDH, F, TCATTTCCTGGTATGACAACGA, R, GTCTTACTCCTTGGAGGCC; and U6, F, ATACAGAGAAAGTTAGCACGG, R, GGAATGCTTCAAAGAGTTGTG. Gene expression was calculated with the 2^−*ΔΔ*Ct^ method with GAPDH as an internal control for lncRNAs and CST1 and U6 for miR-185-5p.

### 2.4. Immunofluorescence

TU686 and TU177 cells were treated with 4% paraformaldehyde for fixation, 0.1% Triton-X for permeabilization, and 5% normal goat serum for blocking for 10 min, 5 min, and 30 min, respectively. Incubation was performed with anti-CST1 primary antibody (1 : 100, ab124281, Abcam, Shanghai, China) and Alexa Fluor® 488-labeled secondary antibody anti-IgG (1 : 200, ab150077, Abcam). Nuclei were costained with 4′,6-diamidino-2-phenylindole (DAPI). An ECLIPSE Ni microscope (Nikon, Japan) was utilized to capture the images.

### 2.5. Western Blotting

Total proteins were extracted with RIPA lysis buffer (ab156034, Abcam, Cambridge, UK), separated using SDS-PAGE, and transferred to a nitrocellulose membrane. After blocking with 5% nonfat milk and 0.1% Tween 20, blots were incubated with anti-CST1 (1 : 1000, ab68329, Abcam) and anti-GAPDH (1 : 10000, ab8245, Abcam) at 4°C overnight. The blots were then incubated with HRP-labeled IgG secondary antibodies and visualized using ECL method with a kit (#P0018FS, Beyotime, Shanghai, China).

### 2.6. Colony Formation Assay

In 6-well plates, transfected TU686 and TU177 cells with 1,000 cells per well were inoculated and cultured in a 60 mm dish at 37°C in 5% CO_2_ for 2 weeks. Cell culture was discontinued once the colonies were visible to the human eye, which were then stained with 1% crystal violet for 20 min (500 *μ*L/well, Beyotime, Shanghai, China). The colonies that contained more than 50 cells were counted using an inverted light microscope (Nikon ECLIPSE Ti, Japan).

### 2.7. Transwell Assay

An 8 *μ*m pore polycarbonate membrane Boyden chamber was used to detect invasion (Corning, USA). Cells (2 × 10^5^) were cultured in serum-free medium in the upper transwell chamber that was precoated with 50 *μ*L of Matrigel. 0.5 mL of complete medium with 10% FBS was added into the bottom chamber. Incubation was performed at 37°C in 5% CO_2_ for one day. Next, cells on the upper chamber were wiped off with a cotton swab. Cells invading onto the lower surface of the membrane were fixed with methanol and stained with 2% crystal violet for 10 min. Five random visual fields of each membrane were selected for counting the invaded cells under a microscope (BHNK-PH001, Nikon Corporation; magnification, 100x). Cell migration assay was done similar to the migration assay except that no Matrigel was used.

### 2.8. Dual Luciferase Assay

The wild-type (Wt) or mutant (Mut) miR-185-5p (Wt: 5′-UGGAGAGAAAGGCAGUUCCUGA-3′, Mut: 5′-UCAGCUAUUAGGCAGUUCCUGA-3′) or CST1 3′UTR sequences (Wt: 5′-UCGGGCUCUCACCCUCCUCUCCU-3′, Mut: 5′-UCGGGCUCUCACCCGAGAUAGGA-3′) were subcloned into the pmirGLO vector (Promega Corporation, Madison, WI, USA) to construct pmirGLO-miR-185-5p-Wt/Mut or pmirGLO-CST1 3′UTR-Wt/Mut plasmids. TU686 and TU177 cells were transfected with the pmirGLO luciferase reporter plasmids and miR-185-5p mimics or sh-LINC01278 at a final concentration of 20 nM. Following 48 h transfection, renilla luciferase activity was normalized to firefly luciferase activity. Relative luciferase activity was calculated the Dual-Luciferase Reporter Assay system (Promega).

### 2.9. Statistical Analysis

Data are expressed as mean ± standard deviation (SD) from three biological repeats and three technical repeats. For comparison among three or more independent groups, analysis of variance followed by Dunnett's post hoc test and Tukey's post hoc test was performed. When comparing two independent groups, independent *t*-test was performed. Data were processed and analyzed with SPSS version 22.0 (SPSS Inc., USA). *P* < 0.05 was considered to indicate a statistically significant difference.

## 3. Results

### 3.1. CST1 Shows Upregulation in Laryngeal Cancer Cells

**Figure 1:**
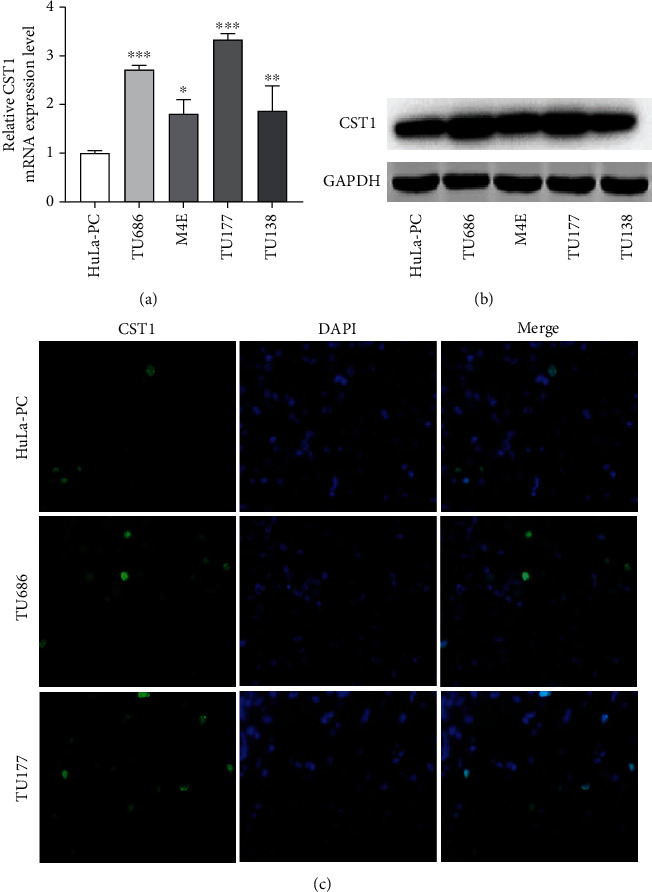
CST1 shows upregulation in laryngeal cancer cells. (a) RT-qPCR analysis of CST1 expression in TU686, M4E, TU177, TU138, and control HuLa-PC cells. (b) Western blotting of CST1 protein in TU686, M4E, TU177, TU138, and control HuLa-PC cells. (c) Immunofluorescence staining of CST1 in TU177, TU138, and control HuLa-PC cells. ^∗^*P* < 0.05, ^∗∗^*P* < 0.01, and ^∗∗∗^*P* < 0.001.

Compared with HuLa-PC cells, CST1 exhibited higher expression in TU686, M4E, TU177, and TU138 cells at the mRNA and protein levels (Figures 1(a) and 1(b)). TU686 and TU177 cells were used for the subsequent assays due to the relatively higher expression of CST1 in the two cells. Results of immunofluorescence staining assay showed that TU686 and TU177 cells contained more CST1 protein than HuLa-PC cells (Figure 1(c)).

### 3.2. CST1 Knockdown Reduced Laryngeal Cancer Cell Proliferation and Motion

**Figure 2:**
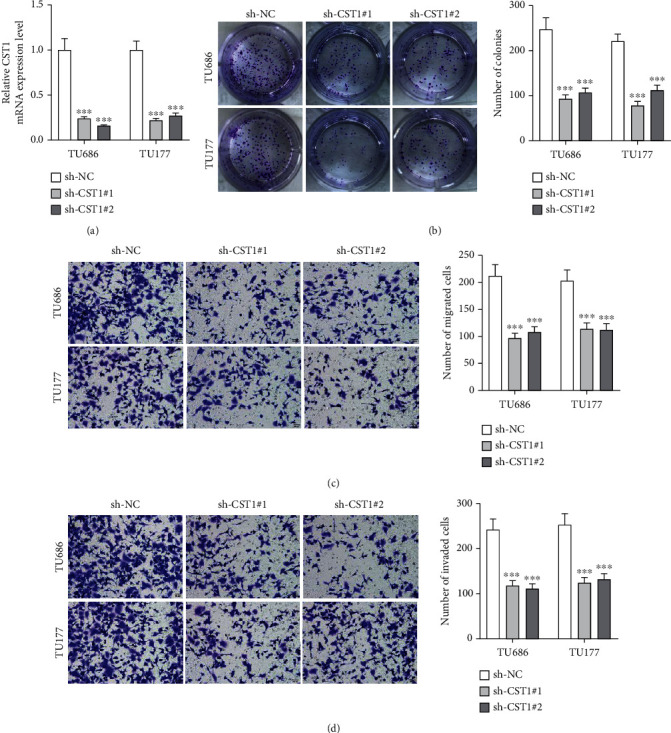
CST1 knockdown reduced laryngeal cancer cell proliferation and motion. (a) RT-qPCR analysis of CST1 expression in TU686 and TU177 cells after transfection with sh-CST1#1/2. (b) Number of colonies formed by sh-CST1#1/2-transfetced TU686 and TU177 cells. (c, d) Migrated and invaded TU686 and TU177 cells after transfection with sh-CST1#1/2 were photographed and counted. ^∗∗∗^*P* < 0.001.

CST1 expression was successfully reduced in TU686 and TU177 cells by transfection with sh-CST1#1/2 (Figure 2(a)). CST1 knockdown decreased number of colonies, migrated cells, and invaded cells (Figures 2(b)–2(d)).

### 3.3. miR-185-5p Binds to CST1 3′UTR and Suppresses Its Expression

**Figure 3:**
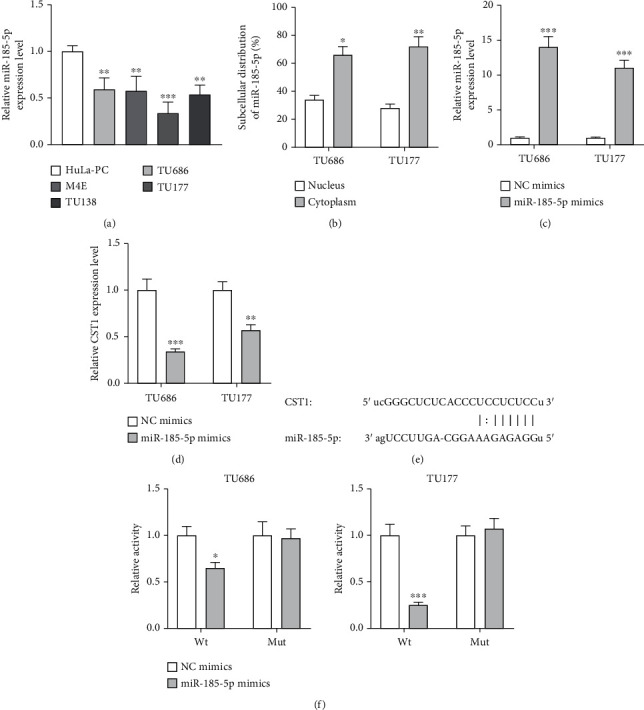
miR-185-5p binds to CST1 3′UTR and suppresses its expression. (a) Expression of miR-185-5p in TU686, M4E, TU177, TU138, and control HuLa-PC cells, as analyzed by RT-qPCR. (b) Expression of miR-185-5p in the nuclear and cytoplasmatic parts of TU686 and TU177 cells, as assessed by RT-qPCR. (c) Overexpression efficiency of miR-185-5p was assessed by RT-qPCR. (d) CST1 expression in miR-185-5p mimic-transfected TU686 and TU177 cells was evaluated by RT-qPCR. (e) Sequences of miR-185-5p and the complementary binding site on CST1 3′UTR. (f) Luciferase activity of pmirGLO-CST1 3′UTR-Wt/Mut in miR-185-5p mimic-transfected TU686 and TU177 cells. ^∗^*P* < 0.05, ^∗∗^*P* < 0.01, and ^∗∗∗^*P* < 0.001.

Based on ENCORI database [16], miR-185-5p was found to bind to CST1 3′UTR (parameter: more than 10 cancer types). miR-185-5p presented lower expression in TU686, M4E, TU177, and TU138 cells (Figure 3(a)) and was distributed more in the cytoplasm than nucleus in TU686 and TU177 cells (Figure 3(b)). Transfection of miR-185-5p mimics successfully enhanced miR-185-5p expression and decreased CST1 expression in TU686 and TU177 cells (Figures 3(c) and 3(d)). A binding site of miR-185-5p on CST1 3′UTR was obtained from ENCORI (Figure 3(e)). miR-185-5p can reduce luciferase activity of pmirGLO-CST1 3′UTR-Wt and had no effects on that of pmirGLO-CST1 3′UTR-Mut (Figure 3(f)).

### 3.4. miR-185-5p Represses Laryngeal Cancer Cell Proliferation and Motion by CST1

**Figure 4:**
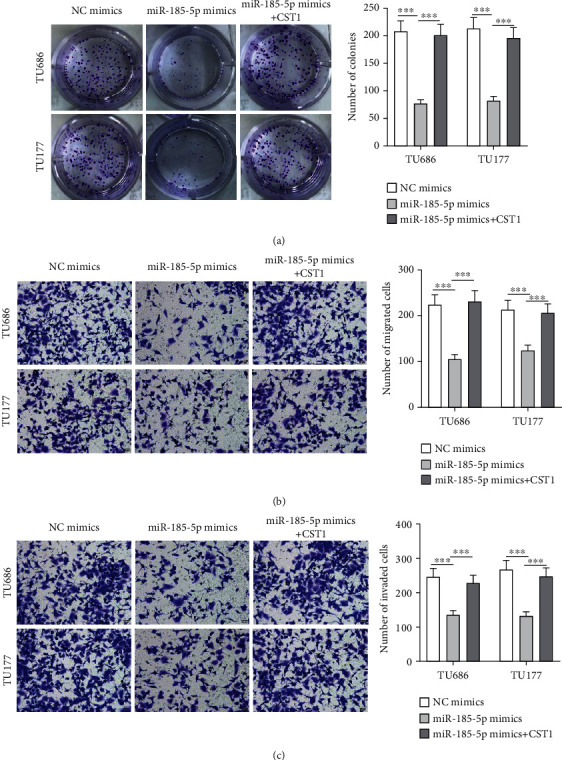
miR-185-5p represses laryngeal cancer cell proliferation and motion by CST1. (a) Number of colonies formed by TU686 and TU177 cells after transfections of NC mimics, miR-185-5p mimics, or cotransfection with miR-185-5p mimics+pcDNA-CST1. (b, c) Migrated and invaded TU686 and TU177 cells after transfections were photographed and counted. ^∗∗∗^*P* < 0.001.

Functions of miR-185-5p in TU686 and TU177 cells were evaluated. It was demonstrated by rescued assays that miR-185-5p mimics decreased number of colonies, migrated cells, and invaded cells, and such effects were rescued by CST1 (Figures 4(a)–4(c)).

### 3.5. LINC01278 Binds with miR-185-5p to Upregulate CST1

**Figure 5:**
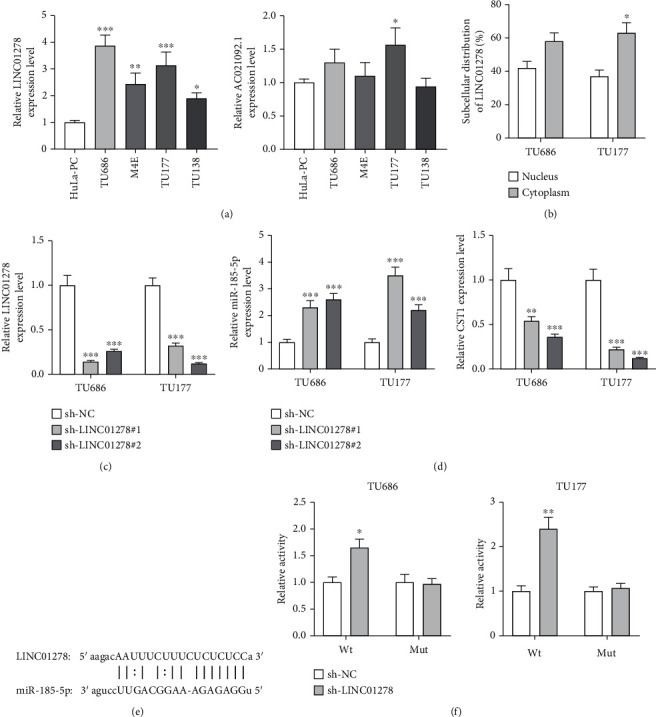
LINC01278 binds with miR-185-5p to upregulate CST1. (a) LINC01278 and AC021092.1 expression in TU686, M4E, TU177, TU138, and control HuLa-PC cells, as analyzed by RT-qPCR. (b) RT-qPCR analysis of LINC01278 expression in the nuclear and cytoplasmatic parts of TU686 and TU177 cells. (c) Knockdown efficiency of LINC01278 was evaluated by RT-qPCR. (d) RT-qPCR analysis of miR-185-5p and CST1 expression in sh-LINC01278#1/2-transfetced TU686 and TU177 cells. (e) Binding site of miR-185-5p on LINC01278. (f) Luciferase activity of pmirGLO-miR-185-5p-Wt/Mut in sh-LINC0127-transfected TU686 and TU177 cells. ^∗^*P* < 0.05, ^∗∗^*P* < 0.01, and ^∗∗∗^*P* < 0.001.

Two lncRNAs, LINC01278 and AC021092.1, were revealed as the upstream molecules of miR-185-5p by ENCORI database (strict stringency in CLIP data and more than 10 cancer types in pan-cancer). LINC01278 is upregulated in TU686, M4E, TU177, and TU138 cells, while AC021092.1 is only upregulated in TU177 cells (Figure 5(a)). Majority of LINC01278 exist in the cytoplasmatic part of TU686 and TU177 cells (Figure 5(b)). LINC01278 was effectively reduced by transfection with sh-LINC01278#1/2 (Figure 5(c)). sh-LINC01278 increased miR-185-5p expression and decreased CST1 expression in TU686 and TU177 cells (Figure 5(d)).  Figure 5(e) exhibited the binding sequences between LINC01278 and miR-185-5p, as obtained from ENCORI. LINC01278 deficiency increased the luciferase activity of pmirGLO-miR-185-5p-Wt and caused no significant effects on that of pmirGLO-miR-185-5p-Mut (Figure 5(f)).

### 3.6. LINC01278 Inhibits TU686 and TU177 Cell Proliferation and Motion by the miR-185-5p/CST1 Axis

**Figure 6:**
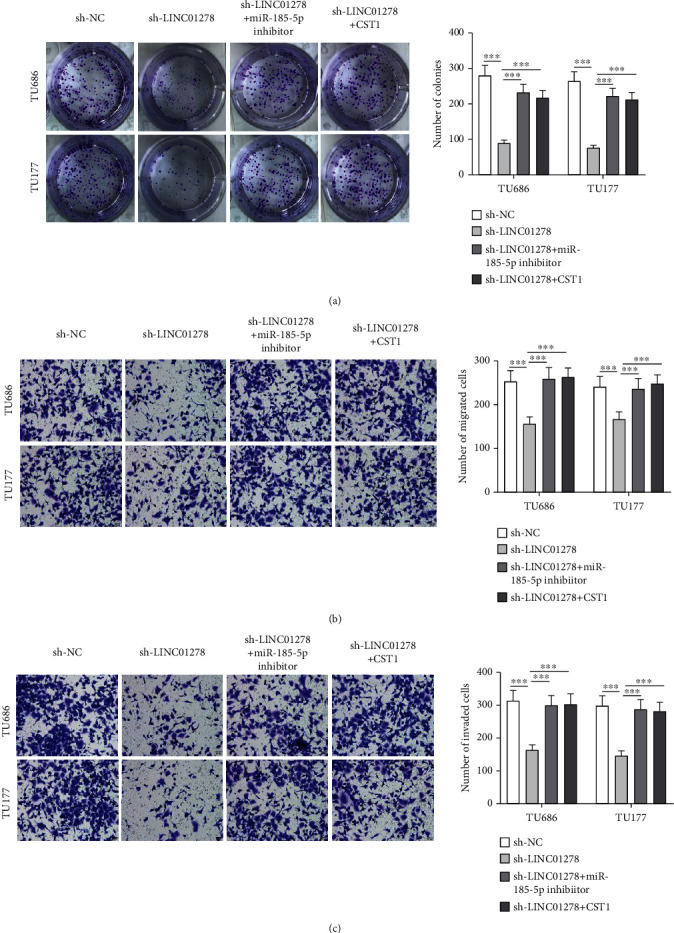
LINC01278 inhibits TU686 and TU177 cell proliferation and motion by the miR-185-5p/CST1 axis. (a) Number of colonies formed by TU686 and TU177 cells after transfections of sh-NC, sh-LINC01278, or cotransfections with sh-LINC01278+miR-185-5p inhibitor, sh-LINC01278+pcDNA-CST1. (b, c) Number of migrated and invaded TU686 and TU177 cells after transfections. ^∗∗∗^*P* < 0.001.

LINC01278 deficiency reduced number of TU686- and TU177-formed colonies and decreased number of migrated cells and invaded cells, and such effects were rescued by miR-185-5p knockdown and CST1 (Figures 6(a)–6(c)).

## 4. Discussion

Understanding of the molecular mechanism of laryngeal cancer pathogenesis is beneficial to the treatment of this disease. CST1 has the potential to promote cancer cell proliferation and motility [14, 17]. CST1 was identified as an upregulated gene in laryngeal cancer tissues based on online data. We confirmed its upregulation in laryngeal cancer cells. Results of biological experiments revealed the inhibitory influence of CST1 deficiency on the proliferative, migrative, and invasive properties of laryngeal cancer cells. Our experimental findings suggested that CST1 could act as an oncogene to mediate laryngeal cancer carcinogenesis. After confirming the role of CST1 in laryngeal cancer cells, the lncRNA–miRNA–CST1 interactions were investigated.

miRNAs participate in multiple activities in cells through modulating protein-coding genes. In recent study, it has been found that the level of miR-185-5p was significantly reduced in laryngeal squamous cell carcinoma tissues and serves as a target gene for specific lncRNAs [18]. For example, Fan et al. [19] demonstrated that miR-185-5p reduces the gene expression of HBV of hepatoma carcinoma cells by inhibiting ELK1. It is also reported that miR-506 inhibits the growth of laryngeal cancer cells by the inhibition of YAP126 [20]. In our study, miR-185-5p was revealed as the regulatory factor for CST1 by bioinformatics analysis and its binding to CST1 3′UTR was confirmed by our experimental results. miR-185-5p shows downregulation in laryngeal cancer, which is consistent with a previous study [18]. We further demonstrated that miR-185-5p elicits a negative effect on laryngeal cancer proliferation and motility by targeting CST1, suggesting the antioncogenic function of miR-185-5p in laryngeal cancer.

Growing evidence showed that lncRNAs play crucial roles in cancers through sponging miRNAs [21, 22]. LINC01278, an oncogene in other cancers, has been found related to clinical staging, distant metastasis, and poor prognosis of patients [23]. It is found that LINC01278 is significantly downregulated in papillary thyroid carcinoma tissues and cell lines and exerts a suppressor function in tumor cells [24]. Huang et al. have found that LINC01278 promotes the metastasis of hepatocellular carcinoma by targeting miR-1258-Smad2/3 [25]. Meanwhile, Qi et al. found that in osteosarcoma tissues, the expression of LINC01278 is enhanced, and it promotes the proliferation of osteosarcoma cells through miR-133a-3p/PTHR1 signal [26]. These two latest studies suggest that the role of LINC01278 in tumor cells may depend on the specific tumor types, tumor microenvironment, or downstream targets. Our study further supports the oncogenic role of LINC01278. We found that LINC01278 serves as the regulatory factor for miR-185-5p. It upregulates CST1 expression by binding with miR-185-5p to antagonize its suppressive effect on CST1. Compared with miR-185-5p, LINC01278 has the opposite effects in laryngeal cancer to facilitate cell proliferation and motility.

A major shortcoming of this research is lacking animal studies to further support the role of LINC01278/miR-185-5p/CST1 in laryngeal cancer at the preclinical level. Clinical data to reveal the expression levels of miR-185-5p and LINC01278 in patients with laryngeal cancer were needed. Moreover, apoptosis is an important factor associated with cancer progression. Our further work will focus on the effects of the LINC01278-mediated ceRNA pattern in the apoptosis or other forms of cell death in laryngeal cancer.

To sum up, LINC01278 is an oncogene in laryngeal cancer by contributing to cancer cell proliferative, migrative, and invasive properties through the miR-185-5p/CST1 axis, which sheds new insight into the ceRNA mechanism of laryngeal cancer pathogenesis.

## Data Availability

The data used to support the findings of this study are available from the corresponding author upon request.
